# Early Clinical and Molecular Detection of Alzheimer's Disease

**DOI:** 10.4061/2011/818639

**Published:** 2011-11-10

**Authors:** Benedetta Nacmias, Christiane Reitz, Thomas Arendt

**Affiliations:** ^1^Department of Neurological and Psychiatric Sciences, University of Florence, Viale Morgagni 85, 50134 Florence, Italy; ^2^Gertrade H. Sergievsky Center, Taub Institute for Research on Alzheimer's Disease and the Aging Brain, and the Department of Neurology, Columbia University, New York, NY 10032, USA; ^3^Paul Flechsig Institute for Brain Research, University of Leipzig, 04109 Leipzig, Germany

## 

Alzheimer's disease (AD) is the most frequent neurodegenerative disease in the elderly. It is characterized by progressive impairment in multiple cognitive domains, in particular memory, leading to severe impairment in daily living followed by progressive physical deterioration and death. The neuropathological hallmark of AD is the presence of cortical intracellular neurofibrillary tangles (NFT) and extracellular *β*-amyloid (A*β*) plaques, which leads to synapse dysfunction, neuronal cell loss, and subsequent brain atrophy. In the past few decades the knowledge of the key pathogenic mechanisms of the disease has improved, but it remains far from being fully understood. Traditionally, the diagnosis of AD has been based on clinical symptoms, and accuracy studies of the current clinical criteria conducted in referral have shown high sensitivity for AD. However, identification of the disease, in particular in the early stages, remains difficult as pathological alterations may be apparent several years before the clear-cut clinical picture. 

There is growing evidence that the use of biomarkers will increase our ability to identify AD earlier in the disease process and with higher accuracy. In addition, biomarkers will help elucidate the underlying biological and molecular changes, improve the detection of patients suitable for specific treatments, research studies or drug trials, and will contribute to a better management of the disease in the clinical practice. In this special issue on early clinical and molecular detection of AD, we have invited manuscripts that specifically address issues related to early and improved diagnosis of AD. 

The first two articles address the usefulness of current biological and neuroimaging markers for the diagnosis of AD, including genetic variation, plasma, serum and CSF biomarkers, brain atrophy measures, functional MRI measures, and amyloid burden evaluated by PiB compound. The third paper reevaluates the usefulness of common clinical rating scales for AD, frontotemporal dementia, and vascular dementia using factor analysis. In the fourth paper, the sociocultural impacts of novel molecular technologies for the early diagnosis of AD are addressed. The paper outlines three steps to assess sociocultural impacts. First, conceptual analysis of the ideas underlying technological developments shows how these technologies redraw the boundary between AD and normal ageing and between biological and social approaches of ageing. Second, scenarios are designed depicting different possible futures of AD diagnosis and societal ways to deal with ageing. Finally, the paper reviews the possibilities for deliberation on the potential sociocultural impacts. 

Manuscripts five and six are original research studies assessing the effect of specific genetic variations on AD and AD endophenotypes. While the study by Swaminathan et al. explores the effect of copy number variation (CNV) derived by genome-wide screening in the ADNI cohort on AD and MCI, the study by Tedde et al. explores the effect of genetic variation in *CDKN2A* and *CDKN2B* on AD. Finally, the last paper assesses the effect of siRNAs silencing on gene expression. While commonly studies use RNA interference (RNAi) to investigate the effect of silencing of specific genes on their expression and effect on cell metabolism, the current paper takes this technology further by assessing the effect of allele-specific silencing of a particular mutant allele, in this case mutation L392V in *PSEN1*.

*Benedetta Nacmias* *Benedetta Nacmias* *Christiane Reitz* *Christiane Reitz* *Thomas Arendt* *Thomas Arendt*

